# Potential Blood DNA Methylation Biomarker Genes for Diagnosis of Liver Fibrosis in Patients With Biopsy-Proven Non-alcoholic Fatty Liver Disease

**DOI:** 10.3389/fmed.2022.864570

**Published:** 2022-03-31

**Authors:** Qing-Feng Sun, Liang-Jie Tang, Ming-Jie Wang, Pei-Wu Zhu, Yang-Yang Li, Hong-Lei Ma, Ou-Yang Huang, Liang Hong, Gang Li, Christopher D. Byrne, Giovanni Targher, Wen-Yue Liu, Yan Lu, Ji-Guang Ding, Ming-Hua Zheng

**Affiliations:** ^1^Department of Infectious Diseases, The Third Affiliated Hospital of Wenzhou Medical University, Wenzhou, China; ^2^NAFLD Research Center, Department of Hepatology, The First Affiliated Hospital of Wenzhou Medical University, Wenzhou, China; ^3^Department of Gastroenterology, Ruijin Hospital, School of Medicine, Shanghai Jiao Tong University, Shanghai, China; ^4^Department of Laboratory Medicine, The First Affiliated Hospital of Wenzhou Medical University, Wenzhou, China; ^5^Department of Pathology, The First Affiliated Hospital of Wenzhou Medical University, Wenzhou, China; ^6^Department of General Practice, Affiliated People’s Hospital, Zhejiang Provincial People’s Hospital, Hangzhou Medical College, Hangzhou, China; ^7^National Institute for Health Research Southampton Biomedical Research Centre, University Hospital Southampton, Southampton General Hospital, Southampton, United Kingdom; ^8^Section of Endocrinology, Diabetes and Metabolism, Department of Medicine, Azienda Ospedaliera Universitaria Integrata Verona, University of Verona, Verona, Italy; ^9^Department of Endocrinology, The First Affiliated Hospital of Wenzhou Medical University, Wenzhou, China; ^10^Department of Endocrinology and Metabolism, Zhongshan Hospital, Fudan University, Shanghai, China; ^11^Institute of Hepatology, Wenzhou Medical University, Wenzhou, China; ^12^Key Laboratory of Diagnosis and Treatment for the Development of Chronic Liver Disease in Zhejiang Province, Wenzhou, China

**Keywords:** NAFLD, liver fibrosis, blood DNA methylation, biomarker, MAFLD

## Abstract

**Background and objective:**

This pilot study aimed to identify potential blood DNA methylation (BDM) biomarker genes for the diagnosis of liver fibrosis in non-alcoholic fatty liver disease (NAFLD).

**Methods:**

We included a total of 16 NAFLD patients with significant (SLF, liver fibrosis stage ≥ 2) and 16 patients with non-significant liver fibrosis (NSLF, fibrosis stages 0–1). The association between BDM and liver fibrosis was analyzed. Genes were selected based on a stepwise-filtering with CpG islands containing significant differentially methylated probes.

**Results:**

The two groups of patients were distinguishable through both t-distributed stochastic neighbor embedding (t-SNE) analysis and unsupervised hierarchical clustering analysis based on their BDM status. BDM levels were significantly higher in the NSLF group than in the SLF group. The methylation levels in the island and shelf regions were also significantly higher in the NSLF group, as well as the methylation levels in the first exon, 3′-untranslated region, body, ExonBnd, non-intergenic region, transcription start site (TSS)1500, and TSS200 regions (all *p* < 0.05). BDM status was associated with greater histological liver fibrosis, but not with age, sex, or other histological features of NAFLD (*p* < 0.05). The methylation levels of the hypomethylated CpG island region of *CISTR*, *IFT140*, and *RGS14* genes were increased in the NSLF group compared to the SLF group (all *p* < 0.05).

**Conclusion:**

BDM may stratify NAFLD patients with significant and non-significant liver fibrosis. The *CISTR*, *IFT140*, and *RGS14* genes are potential novel candidate BDM biomarkers for liver fibrosis and these pilot data suggest further work on BDM biomarkers is warranted.

## Introduction

Non-alcoholic fatty liver disease (NAFLD) has become a public health problem, due to its high prevalence globally (1), and there is now evidence that it is a multisystem disease (2) increasing risk of multiple cardiometabolic disorders, such as cardiovascular disease (3) and type 2 diabetes (4). NAFLD is a complex multifactorial disease involving genetic, metabolic and environmental factors. Over the past decade, research into pathogenesis, diagnosis, and treatment has revealed different aspects of NAFLD, challenging the definition of clinical practice and the accuracy of treatment strategies. Recently, several international experts reached a consensus that the old definition of NAFLD does not reflect current knowledge, suggesting that metabolic dysfunction-associated fatty liver disease (MAFLD) is a more appropriate definition (5–7).

Liver fibrosis, which is typically characterized by the deposition of excess extracellular matrix and loss of hepatocyte function, is frequently associated with NAFLD. Studies have revealed that the severity of liver fibrosis is the strongest prognostic factor for all-cause mortality and the development of liver-related complications in people with NAFLD (8–11). The risk of liver-related mortality also increases exponentially with the severity of liver fibrosis (12). NAFLD patients with significant liver fibrosis (SLF, i.e., those with F stage ≥ 2) are at higher risk of liver-related mortality than those with non-significant liver fibrosis (NSLF; F stage 0–1) (12). Therefore, appropriate interventions should be performed in a timely manner to prevent or delay the development of end-stage liver disease associated with liver fibrosis (4). Currently, liver biopsy is the gold standard for the diagnosis of NAFLD and assessment of the severity of liver fibrosis. However, this procedure is invasive, expensive, and potentially associated with some acute complications. There is, therefore, an unmet need for developing non-invasive and cost-effective tests for diagnosing and staging NAFLD, especially for distinguishing between patients with SLF and those with NSLF (13–19).

DNA methylation adds a methyl group to cytosine in the CpG dinucleotide and is one of the major epigenetic forms of DNA modification. DNA methylation often causes transcriptional silencing of gene expression and alterations in chromosomal instability, cell cycle regulation, viability, differentiation, apoptosis, and signaling pathways (20, 21). Methylated circulating and tissue DNA have emerged as biomarkers or therapeutic targets in many chronic diseases, such as prostate cancer (22), bladder cancer (23), pulmonary fibrosis (24), Alzheimer’s disease (25), and NAFLD (26, 27). A previous study revealed that hypomethylation of DNA in early-stage liver fibrosis is critical for the development and progression of liver fibrosis (28). Hepatic stellate cells are activated by methylation changes (resulting in excessive accumulation of extracellular matrix), which are the hallmark of liver fibrosis (29–32). Plasma DNA methylation of peroxisome proliferator-activated receptor (PPAR-γ) can potentially be used to non-invasively stratify liver fibrosis severity in NAFLD (33). Some key methylated CpG sites from peripheral blood leukocytes might also be used as serum biomarkers to differentiate patients with simple steatosis from those with non-alcoholic steatohepatitis (NASH) (34). A recent study by Lai et al. revealed that patients with NAFLD had decreased hepatic levels of global DNA methylation, a parameter that decreases in parallel with increasing hepatic inflammation grade and disease progression (35). However, to date, blood DNA methylation biomarker genes for the diagnosis of liver fibrosis in NAFLD remain to be identified.

In this pilot study, we investigated the association between blood DNA methylation and advanced or mild liver fibrosis, and identified potential blood DNA methylation biomarker genes for the diagnosis of liver fibrosis in patients with biopsy-confirmed NAFLD. Our findings may provide clues for the development of non-invasive tests to diagnose NAFLD-related liver fibrosis.

## Materials and Methods

### Patients and Samples

Patients with biopsy-proven NAFLD were recruited as described in our previous study (36). These patients were recruited at the First Affiliated Hospital of Wenzhou Medical University from December 2016 to January 2018. Specifically, for this study, we randomly selected 16 patients with high liver fibrosis and 16 patients with low liver fibrosis by liver histological staging. The presence of fibrosis stage ≥ 2 was defined as presence of significant liver fibrosis (SLF), whereas fibrosis stages 0–1 were defined as non-significant (NSLF). The two groups of NAFLD patients were well matched for age and sex. For each patient, a complete medical history was obtained, the blood sample was collected and immediately stored at −80°C until use, and a liver biopsy was performed. Patients who had a prior history of significant alcohol consumption, viral hepatitis, autoimmune hepatitis, and other known causes of chronic liver diseases, or those with incomplete data were excluded from the study (36, 37).

The diagnosis of NAFLD was based on histological abnormalities in the liver biopsy examined with hematoxylin-eosin and Masson’s trichrome staining. All stained biopsy samples were analyzed by two experienced liver pathologists, who were blinded to the clinical and laboratory data of participants. All histologic features were scored according to the NASH Clinical Research Network classification system (38), and the fibrosis stage (1–4) was estimated for each NAFLD specimen (39). The fibrosis stage was scored as follows: no fibrosis, stage 0, perisinusoidal or portal fibrosis, stage 1; perisinusoidal and portal/periportal fibrosis, stage 2; bridging fibrosis, stage 3; highly suspicious or definite cirrhosis, stage 4 (39). The presence of fibrosis stage ≥ 2 was defined as SLF, whereas fibrosis stages 0–1 were defined as NSLF (36, 37).

This study was registered in the Chinese Clinical Trial Registry (ChiCTR-EOC-17013562). The study protocol was approved by the Ethics Committee of The First Affiliated Hospital of Wenzhou Medical University (2016-246, 1 December 2016). Written informed consent was obtained from each participant.

### DNA Extraction and Illumina 850K (EPIC) DNA Methylation Array

Genomic DNA from fresh-frozen blood samples was isolated using the Magnetic Universal Genomic DNA Kit (Tiangen, Beijing, China) according to the manufacturer’s protocol. The Infinium Methylation EPIC BeadChips (Illumina, San Diego, CA, United States) was used to detect bisulfite-converted genomic DNA by hybridization. After scanning (iScan, Illumina), the raw intensity data were preprocessed, normalized, and analyzed with the ChAMP package (2.14.0) (40). Cross-reactive probes, probes with single nucleotide polymorphisms (SNPs) (41), and probes on X or Y chromosomes, as well as all probes with incomplete and non-significant detection *P*-values (*P* > 0.01) were removed. The resulting dataset of autosomal probes was used for subsequent analyses.

### Differential Methylation Analysis

Beta (β) values were calculated to denote the methylation levels at CpG sites. The β value 0.0 represents no methylation and 1.0 represent 100% methylation (42). Mean β values were calculated for moderate to severe liver fibrosis (i.e., the SLF group) and mild or no liver fibrosis (i.e., the NSLF group). The Δβ value was the result of the mean β value of cases minus that of controls. A positive Δβ value indicated relative hypermethylation, and a negative value denoted hypomethylation in blood DNA. The *limma* (3.40.2) package was used to analyze differential methylation (43). Differentially methylated probes (DMPs) were generated and identified after comparing the mean β values between the NSLF and SLF groups for a particular CpG site. The cut-off threshold for significant DMPs was a Benjamini-Hochberg-adjusted *p*-value for multiple comparisons <0.05 and |Δβ| > 0.1.

### t-Distributed Stochastic Neighbor Embedding and Clustering Analysis

To examine whether the blood DNA methylation status was associated with the severity of liver fibrosis, the t-SNE analysis and unsupervised hierarchical clustering analysis of the overall blood DNA methylation status of NAFLD patients with increasing liver fibrosis were performed using the pheatmap package in R V4.1.0.

### Distribution Analysis of Differential Methylated Probes

Differentially methylated probes were classified into different groups according to their distributions of CpG island regions and gene regions. The expected counts were calculated with the probes that remained after filtering. A multinomial goodness-of-fit Chi squared test was performed to analyze the significance of differences between groups. The blood DNA methylation levels and the distributions of DMPs in CpG island regions and gene regions in the SLF and NSLF groups were compared.

### Enrichment of Differentially Methylated Probe Genes in Gene Ontology Terms and Kyoto Encyclopedia of Genes and Genomes Pathways

The genes that contained DMPs were first mapped and identified. The enrichment of these DMP genes in GO terms and KEGG pathways was analyzed using the DAVID bioinformatics tool (44).

### Identification of Candidate Biomarkers From Blood Differentially Methylated Genes for Liver Fibrosis

To identify potential biomarkers from differentially methylated genes for liver fibrosis in the blood, DMPs found at the CpG sites were filtered via the following sequential steps: (1) selection of DMPs with a mean blood DNA methylation level greater than 0.7 that represents extremely high methylation, or less than 0.3 that represents extremely low methylation, because extremely high or low DNA methylation levels are more likely to be associated with abnormal gene expression; (2) selection of DMPs in non-intergenic region (IGR); (3) island and shelf regions in CpG islands; and (4) after applying the above-mentioned filters, the resultant DMPs were mapped to genes. Genes with at least two DMPs were considered candidate biomarkers for liver fibrosis from blood differentially methylated genes. Their methylation levels in the SLF and NSLF groups were subsequently compared.

### Statistical Analysis

Statistical analyses of all data were performed with R (Version 4.1.0). Probes showing significant differences in DNA methylation levels between the NSLF and SLF groups of NAFLD patients were identified using the limma package. A Benjamini-Hochberg-adjusted *p-value* < 0.05 and |Δβ| > 0.1 for multiple comparisons was considered to be statistically significant.

## Results

### Clinical Information for Patients With Liver Fibrosis

A total of 32 selected patients with biopsy-proven NAFLD and varying levels of liver fibrosis were divided into two groups: the SLF group with high liver fibrosis (*n* = 16) and the NSLF group with low liver fibrosis (*n* = 16). As shown in Table 1, the two groups of patients were well matched for age and sex. No significant differences were found in main biochemical parameters, except for higher HbA1c levels in the SLF group, as well as in other histology features of NASH (steatosis, lobular inflammation, or ballooning).

**TABLE 1:** Main clinical and biochemical characteristics of patients with biopsy-proven NAFLD stratified by high or low levels of liver fibrosis.

Levels of liver fibrosis		High (n = 16)	Low (n = 16)	P-value
Age (years)		32.31 ± 8.20	33.75 ± 6.82	0.59
Sex (*n*, %)	Men	13 (81.2)	14 (87.5)	1.00
	Women	3 (18.8)	2 (12.5)	
Body mass index (kg/m^2^)		28.72 ± 3.9	27.31 ± 3.1	0.27
Waist circumference (cm)		95.50 ± 9.6	92.56 ± 6.9	0.33
Fibrosis stage (*n*, %)	0	0 (0.0)	3 (18.8)	<0.001*
	1	0 (0.0)	13 (81.2)	
	2	12 (75.0)	0 (0.0)	
	3	3 (18.8)	0 (0.0)	
	4	1 (6.2)	0 (0.0)	
NAS steatosis (*n*, %)	0	1 (6.2)	1 (6.2)	0.20
	1	6 (37.5)	4 (25.0)	
	2	1 (6.2)	6 (37.5)	
	3	8 (50.0)	5 (31.2)	
NAS hepatocellular ballooning (*n*, %)	0	1 (6.2)	2 (12.5)	0.72
	1	9 (56.2)	7 (43.8)	
	2	6 (37.5)	7 (43.8)	
NAS lobular inflammation (*n*, %)	1	11 (68.8)	12 (75.0)	1.00
	2	5 (31.2)	4 (25.0)	
Glucose (mmol/L)		4.84 ± 0.63	5.36 ± 0.97	0.08
HbA1c (%)		6.20 ± 1.36	5.41 ± 0.27	0.03*
Insulin (pmol/L)		122.25 ± 63.09	290.18 ± 492.64	0.19
C-Peptide (pmol/L)		1055.47 ± 370.62	1498.88 ± 1339.00	0.21
Total cholesterol (mmol/L)		5.13 ± 0.90	5.03 ± 1.31	0.80
Triglycerides (mmol/L)		2.06 ± 1.00	2.51 ± 1.51	0.33
HDL-cholesterol (mmol/L)		1.02 ± 0.10	0.99 ± 0.17	0.45
LDL-cholesterol (mmol/L)		3.21 ± 0.87	3.06 ± 0.97	0.64
Total bilirubin (μmol/L)		19.12 ± 8.11	16.31 ± 6.45	0.29
Albumin (g/L)		47.67 ± 3.12	48.58 ± 3.58	0.45
Alanine aminotransferase (U/L)		107.75 ± 86.28	160.44 ± 156.93	0.25
Aspartate aminotransferase (U/L)		83.56 ± 73.71	75.56 ± 54.80	0.73
Alkaline phosphatase (U/L)		81.69 ± 16.52	89.75 ± 20.20	0.23
Gamma-glutamyltransferase (U/L)		77.94 ± 59.23	79.62 ± 39.62	0.93
Serum creatinine (μmol/L)		65.44 ± 12.72	67.50 ± 12.85	0.65
eGFR (ml/min/1.73 m^2^)		127.45 ± 16.14	125.84 ± 14.48	0.77
Uric acid (μmol/L)		464.19 ± 109.70	448.62 ± 120.35	0.71

### The Blood DNA Methylation Status in the Significant Liver Fibrosis and Non-significant Liver Fibrosis Groups

**FIGURE 1:**
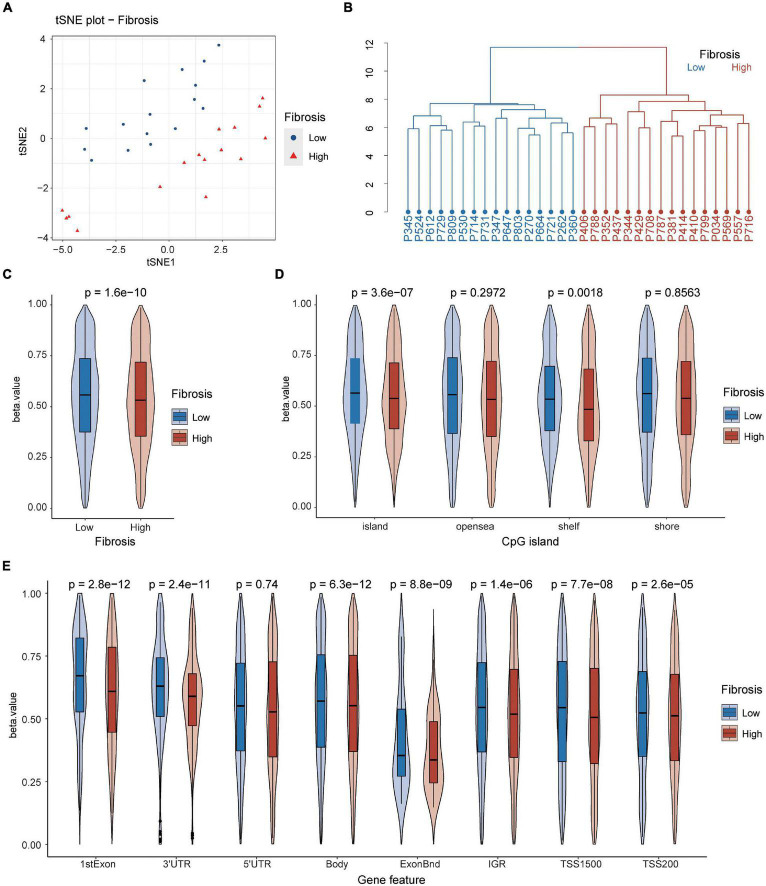
The blood DNA methylation status in NAFLD patients with different levels of liver fibrosis. The SLF and NSLF groups of NAFLD patients were stratified with the t-SNE analysis **(A)** and the unsupervised hierarchical clustering analysis **(B)** based on the overall blood DNA methylation status. **(C)** The methylation levels (i.e., the beta value) of blood DNA in the SLF and NSLF groups. **(D,E)** Comparison of the methylation levels of different CpG island regions **(D)** and different gene regions **(E)** of blood DNA in the SLF and NSLF groups. SLF, significant liver fibrosis; NSLF, non-significant liver fibrosis.

To examine whether the blood DNA methylation status was associated with greater liver fibrosis, we performed the t-SNE analysis and unsupervised hierarchical clustering analysis of the overall blood DNA methylation status amongst the two groups of NAFLD patients. Our results show that the SLF and NSLF groups were significantly different through both the t-SNE analysis (Figure 1A) and the unsupervised hierarchical clustering analysis (Figure 1B) based on the blood overall DNA methylation status.

The blood DNA methylation levels (i.e., the beta value) (Figure 1C) and the methylation levels in the island and shelf regions (Figure 1D) were significantly higher in the NSLF group than in the SLF group. The methylation levels in the first exon, 3′-untranslated region (UTR), body, ExonBnd, IGR, transcription start site (TSS)1500, and TSS200 regions were also significantly higher in the NSLF group Figure 1E). Conversely, there were no significant differences in the open sea and shore regions (Figure 1D) or the 5′-UTR region (Figure 1E) between the two groups of patients.

### Blood Differentially Methylated Probes in Patients With Diagnosed Liver Fibrosis

**FIGURE 2:**
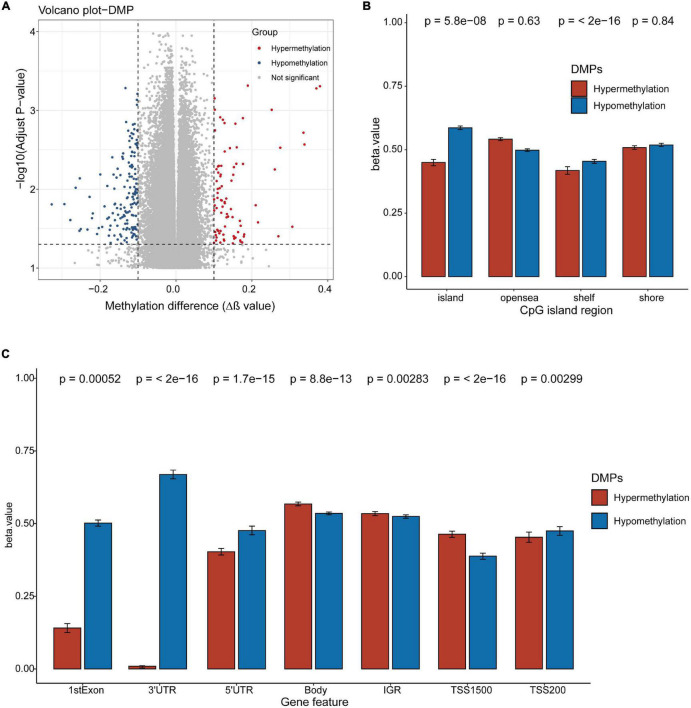
Differentially methylated probes detected in the blood DNA of NAFLD patients with diagnosed liver fibrosis. **(A)** DNA methylation sites (probes) in the blood DNA of NAFLD patients with diagnosed liver fibrosis. Dots, DNA methylation probes. The cutoff values for differentially methylated probes (DMPs): adjusted *p* < 0.05 and |Δβ| > 0.1. Red dots, hypermethylated DMPs; blue dots, hypomethylated DMPs. **(B,C)** The DMPs in CpG island regions **(B)** and gene regions **(C)**. hypermethylated DMPs (red), and hypomethylated DMPs (blue). *P-*values were calculated with the Chi-squared tests.

We identified a total of 233 DMPs in blood samples from our NAFLD patients with varying levels of liver fibrosis (Figure 2A). Both hypomethylated and hypermethylated DMPs were significantly enriched in the CpG island (Figure 2B) and gene regions (Figure 2C). Hypomethylated DMPs were over-represented in the island and shelf regions (Figure 2B), as well as in the first exon, 3′-UTR, 5′-UTR, and TSS200 regions (Figure 2C). Conversely, hypermethylated DMPs were markedly under-represented in the first exon and 3′-UTR regions (Figure 2C), and hypermethylated DMPs were over-represented in the body, IGR, and TSS1500 regions (Figure 2C).

### Enrichment of Differentially Methylated Probe Genes in Gene Ontology Terms and Kyoto Encyclopedia of Genes and Genomes Pathways

**FIGURE 3:**
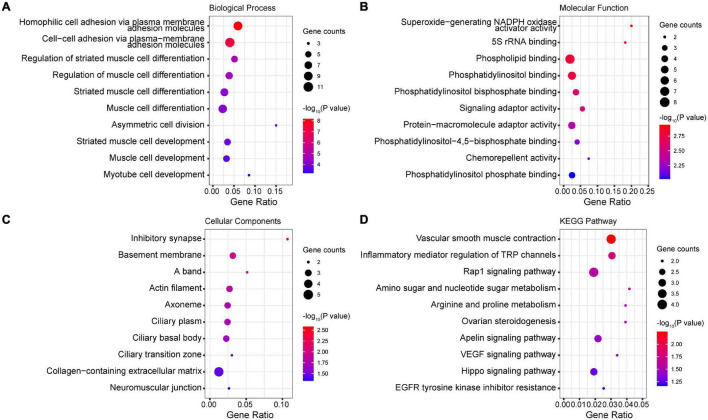
Enrichment of DMP genes in GO terms and KEGG pathways. **(A)** Top enriched GO biological process terms. **(B)** Top enriched GO molecular function terms. **(C)** Top enriched GO cellular components terms. **(D)** Top enriched KEGG pathways.

We mapped the genes that hosted DMPs and analyzed the enrichment of DMP genes in GO terms and KEGG pathways. Our results show that hemophilic and cell-cell adhesion, as well as muscle cell differentiation and development, were the top enriched GO biological process terms (Figure 3A). Additionally, superoxide-generating nicotinamide adenine dinucleotide phosphate (NADPH) oxidase activator activity, 5S rRNA binding, phospholipid binding, and phosphatidylinositol-related binding activity were the top enriched GO molecular function terms (Figure 3B). Under GO cellular components, inhibitory synapse, basement membrane, A band, actin filament, cilia-related structures, extracellular matrix (ECM), and neuromuscular junction were the top enriched terms (Figure 3C). Finally, vascular smooth muscle contraction, inflammatory mediator regulation of transient receptor potential (TRP) channels, signaling pathways such as Rap1, Apelin, Hippo, and vascular endothelial growth factor (VEGF), and amino and nucleotide sugar metabolism were the top enriched KEGG pathways (Figure 3D).

### Association of Blood DNA Methylation With Liver Fibrosis

**FIGURE 4:**
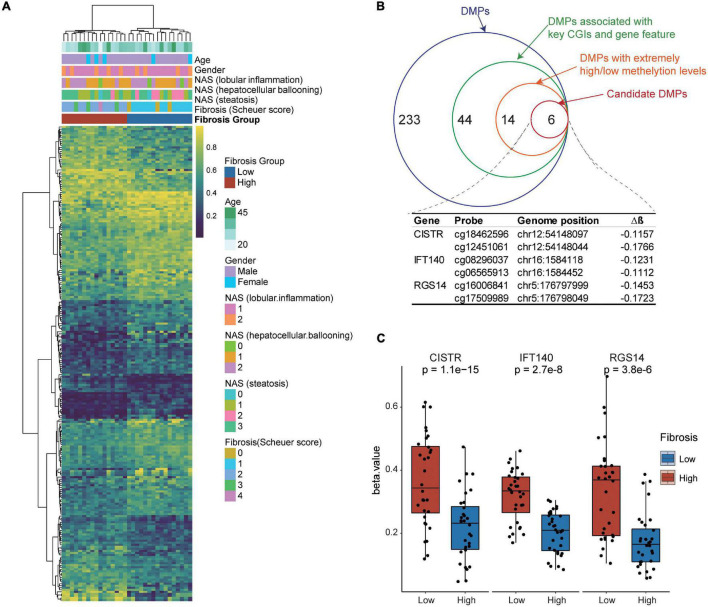
Identification of candidate biomarkers from blood differentially methylated genes for liver fibrosis. **(A)** Association of DNA methylation and clinical features with liver fibrosis. The unsupervised hierarchical cluster analysis of DMPs was performed. DMPs in CpG islands are presented in the rows and independent samples are presented in the columns. The colors in the cells represent methylation level. Blue, unmethylated; red, fully methylated. **(B)** Step-wise selection of candidate DMPs for liver fibrosis. Three genes, i.e., *CISTR*, *IFT140*, and *RGS14*, each harboring at least two DMPs were finally selected. **(C)** The methylation levels in the CpG island regions of blood *CISTR*, *IFT140*, and *RGS14* genes in the NSLF group were significantly higher than those in the SLF group.

We examined the association of blood DNA methylation with liver fibrosis and other clinical features using the unsupervised hierarchical clustering analysis. Our results show that the blood DNA methylation status was significantly associated with greater severity of liver fibrosis, but not with age, sex, and other histologic features of NASH (lobular inflammation, hepatocellular ballooning, or steatosis) (Figure 4A).

### Identification of Candidate Biomarkers From Blood Differentially Methylated Genes for Liver Fibrosis

To identify candidate biomarkers from blood differentially methylated genes for liver fibrosis, we performed a step-wise data-filtering in terms of mean methylation, gene features, CpG islands, and number of DMPs in the genes. We identified a total of six hypomethylated DMPs mapped to the chondrogenesis-associated transcript (*CISTR*), intraflagellar transport complex A (*IFT140*), and regulator of G-protein signaling 14 (*RGS14*) genes (Figure 4B). The methylation levels in the CpG island regions of the *CISTR*, *IFT140*, and *RGS14* genes in the NSLF group were significantly higher than those in the SLF group (Figure 4C).

## Discussion

In this pilot study involving Chinese individuals with biopsy-proven NAFLD, we showed for the first time that histologically classified SLF and NSLF patient groups can be accurately distinguished via the t-SNE analysis and unsupervised hierarchical clustering analysis, based on their blood overall DNA methylation status. The blood DNA methylation levels and the methylation levels in the island and shelf regions were significantly greater in the NSLF group than in the SLF group, but they did not differ between men and women, and were not associated with age, NAS lobular inflammation, NAS hepatocellular ballooning, or NAS steatosis. Consistent with previous results presented by Murphy et al. human genes in the livers with advanced fibrosis were generally hypomethylated relative to those in the livers with mild fibrosis (45). It is known that DNA methylation is a critical process for modulating the activity of hepatic stellate cells, which are essential for fibrogenesis (29–31, 46); so consistent with previous studies, our findings suggest that blood DNA methylation can differentiate NAFLD patients with SLF and NSLF (34, 35). Additionally, we have confirmed and extended previous findings showing that the DNA methylation levels in the island and shelf regions were higher in the NSLF group. We have also shown that the methylation levels in the first exon, 3′-UTR, body, ExonBnd, IGR, TSS1500, and TSS200 regions were significantly higher in the NSLF group. These data suggest that more severe liver fibrosis is characterized by lower levels of blood DNA methylation, which is in agreement with the results from the studies by Komatsu et al. (28) and Lai et al. (35). Thus, it is likely that blood DNA methylation is a potential biomarker for NAFLD-related liver fibrosis and for stratification of SLF and NSLF.

Non-alcoholic fatty liver disease development is influenced by genetic susceptibility and heritability explains inter-individual and ethnic differences of NAFLD development and progression to fibrosis (47). Romeo et al. documented an interplay between the patatin-like phospholipase domain containing 3 (*PNPLA3*) variant I148M and advanced fibrosis in NASH patients (48). Another gene found by genome-wide association studies to be a risk factor for NAFLD, is the trans-membrane 6 superfamily member 2 (TM6SF2), non-synonymous variant rs58542926 (49). Dongiovanni et al. showed that carriers of this genetic variant had lower plasma lipids and more severe steatosis and fibrosis than non-carriers (50). Other genetic variants potentially implicated in NAFLD development and progression were neurocan (NCAN-rs2228603), protein phosphatase 1, regulatory (inhibitor) subunit 3B (PPP1R3B-rs4240624), glucokinase regulator (GCKR-rs780094), and lysophospholipase-like 1 (LYPLAL1-rs12137855) (51).

Previous studies have also shown that plasma DNA methylation of PPAR-γ can potentially be used for non-invasive detection of liver fibrosis severity in NAFLD (33). Additionally, peripheral blood leukocyte *ACSL4*, *CRLS1*, *CTP1A*, *SIGIRR*, *SSBP1*, and *ZNF622* genes containing DMPs might be used as serum biomarkers to stratify NAFLD patients into groups with simple hepatic steatosis and NASH (34). In the current study, we found that the *CISTR*, *IFT140*, and *RGS14* genes were hypomethylated in the island and shelf regions of the CpG island. The methylation levels of blood *CISTR*, *IFT140*, and *RGS14* genes were also significantly higher in the NSLF group than in the SLF group. The hypomethylation status of these genes in NAFLD patients with liver fibrosis was consistent with the findings by Komatsu et al. (28) and Lai et al. (35). Therefore, the *CISTR*, *IFT140*, and *RGS14* genes may be potential novel candidate blood methylation biomarkers for the diagnosis of liver fibrosis in NAFLD.

The *RGS14* gene encodes a multi-functional nucleocytoplasmic shuttling signaling protein involved in modulating microtubule dynamics and spindle formation (46). RGS14 acts as a scaffolding protein that integrates the G protein, Ras/extracellular signal-regulated kinase (ERK), and calcium/calmodulin signaling pathways essential for spine plasticity and cell signaling (52); RGS14 may also reduce hepatic ischemia–reperfusion injury mainly through its interaction with TAK1 and the JNK/p38 signaling axis (53). IFT140 is a key component of IFT complex A, which is responsible for retrograde transportation in cilia. IFT140 is essential in promoting dentin formation and repair, (54) as well as chondrogenic and osteogenic differentiation during bone development (55). The distinctive monoallelic phenotype causes liver cysts (56). The *CISTR* gene is evolutionarily conserved and involved in mesenchymal and prechondrogenic development (57). The roles of the *CISTR*, *IFT140*, and *RGS14* genes in the onset of liver fibrosis in NAFLD patients and their roles in the blood and the relationship with liver fibrosis remain unclear. Therefore, these genes require further validation as potential candidate blood methylation biomarkers for the diagnosis of NAFLD-related liver fibrosis through in-depth progression, limiting or promoting its progression (58). The challenge now is to better understand epigenetic mechanisms and their interactions; these experimental studies could provide further insights into the pathogenesis of chronic liver disease (59). Our analysis has shown that genes containing DMPs in the blood DNA of patients with NAFLD are enriched in hemophilic and cell-cell adhesion, muscle cell differentiation and development under GO biological process terms. Additionally, superoxide-generating NADPH oxidase activator activity, 5S rRNA binding, phospholipid binding, and phosphatidylinositol-related binding activity were enriched under GO molecular function terms. These genes were also enriched in inhibitory synapse, basement membrane, A band, actin filament, ciliary related structures, ECM, and neuromuscular junction under GO cellular components terms. Vascular smooth muscle contraction, inflammatory mediator regulation of TRP channels, signaling pathways such as Rap1, Apelin, Hippo, and VEGF, and amino and nucleotide sugar metabolism were also affected according to KEGG pathway analysis. Collectively, these findings suggest that the blood DNA methylation biomarker genes that are modified with NAFLD-related liver fibrosis are multi-functional genes.

Our pilot study has some important limitations. Firstly, the sample size of the study was small and these findings need to be further validated by larger studies in other ethnic groups. In addition, some *in vitro* cell and *in vivo* animal experiments are now ongoing to further study whether the corresponding gene methylation plays a role in the development and progression of liver fibrosis. Secondly, our study design was cross-sectional and cannot prove causality. Since the DNA methylation profiles of hemostatic genes may not necessarily correlate between liver tissue and peripheral blood in individuals (60), the genes hosting DMPs in the blood DNA of these patients need further investigation.

## Conclusion

The results of our pilot study show that blood DNA methylation markers may allow identification of NAFLD patients with SLF and NSLF. The *CISTR*, *IFT140*, and *RGS14* genes are likely novel candidate blood methylation biomarkers for the diagnosis of liver fibrosis in NAFLD. Genes such as *CISTR*, *IFT140*, and *RGS14* that contain DMPs in the blood DNA of NAFLD patients may provide clues to investigate the role of DNA methylation in the progression of liver fibrosis. Our findings provide new insights into the development of better non-invasive tests for diagnosing liver fibrosis in NAFLD. Further studies are needed to validate these results in independent cohorts in different ethnic groups.

## Data Availability Statement

The data presented in the study are deposited at https://doi.org/10.5281/zenodo.6240813.

## Ethics Statement

The studies involving human participants were reviewed and approved by the Ethics Committee in Clinical Research (ECCR) of The First Affiliated Hospital of Wenzhou Medical University. The patients/participants provided their written informed consent to participate in this study.

## Author Contributions

Q-FS, W-YL, YL, and M-HZ: study concept and design. P-WZ, H-LM, O-YH, LH, and GL: acquisition of data. Y-YL: pathology analysis. L-JT: drafting of the manuscript. GT and CB: critical revision. M-JW: statistical analysis. M-HZ and J-GD: study supervision. All authors contributed to the manuscript for important intellectual content and approved the submission.

## Conflict of Interest

The authors declare that the research was conducted in the absence of any commercial or financial relationships that could be construed as a potential conflict of interest.

## Publisher’s Note

All claims expressed in this article are solely those of the authors and do not necessarily represent those of their affiliated organizations, or those of the publisher, the editors and the reviewers. Any product that may be evaluated in this article, or claim that may be made by its manufacturer, is not guaranteed or endorsed by the publisher.

## Abbreviations

NAFLD: non-alcoholic fatty liver disease
DMPs: differentially methylated probes
SLF: significant liver fibrosis
NSLF: non-significant liver fibrosis
t-SNE: t-distributed stochastic neighbor embedding
UTR: untranslated region
IGR: intergenic region
TSS: transcription start site
NAS: NAFLD activity score
PPAR-γ: peroxisome proliferator-activated receptor
NASH: non-alcoholic steatohepatitis
SNPs: single nucleotide polymorphisms
GO: Gene Ontology
KEGG: Kyoto Encyclopedia of Genes and Genomes
NADPH: nicotinamide adenine dinucleotide phosphate
ECM: extracellular matrix
TRP: transient receptor potential
VEGF: vascular endothelial growth factor
ERK: extracellular signal-regulated kinase.
